# The iNKT cell ligand α-GalCer prevents murine septic shock by inducing IL10-producing iNKT and B cells

**DOI:** 10.3389/fimmu.2024.1457690

**Published:** 2024-09-17

**Authors:** Yun Hoo Park, Sung Won Lee, Tae-Cheol Kim, Hyun Jung Park, Luc Van Kaer, Seokmann Hong

**Affiliations:** ^1^ Department of Integrative Bioscience and Biotechnology, Institute of Anticancer Medicine Development, Sejong University, Seoul, Republic of Korea; ^2^ Department of Biomedical Laboratory Science, College of Health and Biomedical Services, Sangji University, Wonju, Republic of Korea; ^3^ Department of Pathology, Microbiology and Immunology, Vanderbilt University School of Medicine, Nashville, TN, United States

**Keywords:** α-GalCer, iNKT cells, sepsis, B cells, IL10

## Abstract

**Introduction:**

α-galactosylceramide (α-GalCer), a prototypical agonist of invariant natural killer T (iNKT) cells, stimulates iNKT cells to produce various cytokines such as IFNγ and IL4. Moreover, repeated α-GalCer treatment can cause protective or pathogenic outcomes in various immune-mediated diseases. However, the precise role of α-GalCer-activated iNKT cells in sepsis development remains unclear. To address this issue, we employed a lipopolysaccharide (LPS)/D-galactosamine (D-GalN)-induced murine sepsis model and two alternative models.

**Methods:**

Sepsis was induced in wild-type (WT) C57BL/6 (B6) mice by three methods (LPS/D-GalN, α-GalCer/D-GalN, and cecal slurry), and these mice were monitored for survival rates. WT B6 mice were intraperitoneally injected with α-GalCer or OCH (an IL4-biased α-GalCer analog) one week prior to the induction of sepsis. To investigate the effects of α-GalCer-mediated iNKT cell activation on sepsis development, immune responses were analyzed by flow cytometry using splenocytes and liver-infiltrating leukocytes. In addition, a STAT6 inhibitor (AS1517499) and an IL10 inhibitor (AS101) were employed to evaluate the involvement of IL4 or IL10 signaling. Furthermore, we performed B cell adoptive transfers to examine the contribution of α-GalCer-induced regulatory B (Breg) cell populations in sepsis protection.

**Results:**

*In vivo* α-GalCer pretreatment polarized iNKT cells towards IL4- and IL10-producing phenotypes, significantly attenuating LPS/D-GalN-induced septic lethality in WT B6 mice. Furthermore, α-GalCer pretreatment reduced the infiltration of immune cells to the liver and attenuated pro-inflammatory cytokine production. Treatment with a STAT6 inhibitor was unable to modulate disease progression, indicating that IL4 signaling did not significantly affect iNKT cell-mediated protection against sepsis. This finding was confirmed by pretreatment with OCH, which did not alter sepsis outcomes. However, interestingly, prophylactic effects of α-GalCer on sepsis were significantly suppressed by treatment with an IL10 antagonist, suggesting induction of IL10-dependent anti-inflammatory responses. In addition to IL10-producing iNKT cells, IL10-producing B cell populations were significantly increased after α-GalCer pretreatment.

**Conclusion:**

Overall, our results identify α-GalCer-mediated induction of IL10 by iNKT and B cells as a promising option for controlling the pathogenesis of postoperative sepsis.

## 1 Introduction

Sepsis is a critical condition that poses a significant risk to one’s life. It develops as an immune response to severe infection, causing a dysregulated hyperinflammatory reaction within the body. If multiple organs fail acutely, the condition progresses to septic shock. Unfortunately, sepsis is a frequent cause of death among critically ill patients, contributing to one in three deaths of hospitalized patients (1). During sepsis, various pathogen-derived molecules such as lipopolysaccharide (LPS) and lipoteichoic acid can induce pro-inflammatory cytokines such as IL12 and IL1β by hepatic macrophages that, in turn, can activate a variety of other cell types such as natural killer (NK) cells, ultimately leading to septic shock (2). Like Gram-negative bacteria-derived LPS, Gram-positive bacteria (e.g., group B *Streptococci*)-derived glycolipids can induce septic shock in a manner dependent on the T cell receptor (TCR)-mediated activation of CD1d-restricted invariant natural killer T (iNKT) cells (3). Activated iNKT cells can facilitate liver injury during septic shock by secreting type 1 cytokines such as IFNγ and TNFα (4, 5). While LPS plus 2-amino-2-deoxy-D-galactose (D-galactosamine [D-GalN]) co-injection mimics Gram-negative bacteria-induced sepsis, glycolipid alpha-galactosylceramide (α-GalCer) plus D-GalN can induce iNKT cell-mediated sepsis mimicking the sepsis elicited by Gram-positive bacteria-derived glycolipids (4, 6).

iNKT cells are a unique T cell subset recognizing glycolipids, presented by the non-classical MHC molecule CD1d. Moreover, iNKT cells can be divided into iNKT1 (PLZF^low^T-bet^hi^ and IFNγ), iNKT2 (PLZF^hi^GATA3^hi^ and IL4), or iNKT17 (PLZF^int^RORγt^hi^ and IL17) cells based on their transcription factor and cytokine profiles (7). Upon stimulation, iNKT cells can release both pro-inflammatory cytokines (e.g., IFNγ, TNFα, and IL6) and anti-inflammatory cytokines (e.g., IL4, IL10, IL22, and TGFβ) (8, 9). As iNKT cells rapidly produce various cytokines following TCR stimulation, these cells can initiate acute immune responses and sustain immune responses, leading to chronic states of immune cell activation (4, 10, 11). In addition, another subset of iNKT cells, IL10-producing iNKT10 cells characterized by the expression of the E4BP4 transcription factor, is predominantly located in the white adipose tissue and displays anti-inflammatory functions (12).

IL10-producing B cells, also known as regulatory B (Breg) cells, release soluble immunomodulatory molecules (e.g., adenosine and indoleamine 2,3-dioxygenase [IDO]) as well as anti-inflammatory cytokines (e.g., IL35 and TGFβ). Thus, it has been suggested that these cells play a vital role in controlling autoimmunity (13, 14). Moreover, CD1d-expressing B cells can ameliorate arthritis by inducing the differentiation of iNKT cells with immune suppressive effector functions (15). In particular, it has been reported that IL10-producing B cells display notable therapeutic potential in the endotoxin-induced septic shock model (16).

α-GalCer, a glycolipid derived from the marine sponge *Agelas mauritianus*, has been identified as a potent ligand for both human and mouse iNKT cells. Due to its potent immune-modulating properties, α-GalCer shows therapeutic efficacy against immune-related disorders, including cancer, microbial infections, autoimmune diseases, and inflammatory conditions (17). Furthermore, repeated *in vivo* α-GalCer stimulation can induce profound iNKT cell anergy and differentiation into iNKT10 cells (18–20). Previous studies have reported that α-GalCer-mediated induction of iNKT10 cells can be sustained for a month, and these iNKT10 cells thereby significantly influenced immune responses such as tumor progression and experimental autoimmune encephalomyelitis (EAE) (18, 21). Although iNKT cells have been implicated in the severity of inflammatory diseases, the role of α-GalCer-activated iNKT cells in sepsis has yet to be fully elucidated. Therefore, in this study, we investigated the effects of α-GalCer-mediated pre-activation of iNKT cells on sepsis.

## 2 Materials and methods

### 2.1 Mice and reagents

Wild-type (WT) C57BL/6 (B6) mice were purchased from Jung Ang Lab Animal Inc. (Seoul, Republic of Korea). IL4/GFP cytokine reporter (4Get) mice were kindly provided by Dr. R. Locksley (University of California, San Francisco, CA, USA). All mice used in this study were on a B6 genetic background, maintained at Sejong University, and used for experiments at 6-12 weeks of age. Mice were maintained on a 12-hour light/12-hour dark cycle in a temperature-controlled barrier facility with free access to food and water. Mice were fed a γ-irradiated sterile diet and provided with autoclaved tap water. Age- and sex-matched mice were used for all experiments in this study. The animal experiments were approved by the Institutional Animal Care and Use Committee of Sejong University (SJ-20210704E1). Alpha-GalCer was purchased from Enzo Life Sciences (Farmingdale, NY, USA). LPS derived from *Escherichia coli* (serotype 0111:B4) was purchased from Sigma-Aldrich (St. Louis, MO, USA). STAT6 inhibitor (AS1517499, Cat. #HY-100614) and IL10 inhibitor (AS101, Cat. #2446/10) were purchased from MedChemExpress (Monmouth Junction, NJ, USA) and Tocris Bioscience (Bristol, UK), respectively.

### 2.2 *In vivo* treatment of mice with α-GalCer or OCH

For activation of iNKT cells with α-GalCer or OCH, mice were injected intraperitoneally (i.p.) with α-GalCer (2 μg/mouse) or OCH (2 μg/mouse) dissolved in phosphate-buffered saline (PBS). Littermates injected with PBS only were used as a negative control.

### 2.3 Induction of septic shock

Mice were injected i.p. with α-GalCer (2 µg/mouse) or Vehicle (Veh). Seven days later, septic shock was induced by three different methods, as follows: First, for LPS/D-GalN-induced septic shock, mice were injected i.p. with LPS (2 µg/mouse) plus D-GalN (25 mg/mouse). Second, for α-GalCer/D-GalN-induced septic shock, mice were injected i.p. with α-GalCer (2 µg/mouse) plus D-GalN (10 mg/mouse) (4). After the challenge, all animals were monitored to evaluate sepsis-induced lethality for 72 hours. Third, for cecal slurry (CS)-induced sepsis, CS was prepared from the cecal feces of WT B6 male mice, as described by Starr et al. (22). Briefly, cecal feces was diluted with water and subsequently filtered with the first 860 μm and second 190 μm screen mesh (Bellco Glass, Inc., Vineland, NJ, USA) to remove fecal debris, then resuspended in 15% glycerol at 100 mg/ml. Aliquots were stored at -80°C until ready for use. Mice were injected i.p. with CS (1.3 mg feces/g body weight) to induce sepsis. All animals were monitored to evaluate sepsis-induced lethality for 48 hours after the challenge.

### 2.4 Injection of STAT6 or IL10 inhibitors

To evaluate whether IL4 or IL10 signaling are involved in α-GalCer-mediated preventive effects on sepsis, mice were injected with either a STAT6 inhibitor (AS1517499, 10 mg/kg body weight) or an IL10 inhibitor (AS101, 10 μg/mouse) every other day or daily for one week, respectively, starting from α-GalCer (2 µg/mouse) pretreatment until sepsis induction.

### 2.5 Isolation of liver leukocytes

Mice were anesthetized using a combination of ketamine (40 mg/kg) and xylazine (4 mg/kg). They were perfused via the left heart ventricle with cold and sterile PBS for 3 min to remove peripheral blood mononuclear cells (PBMCs) from the blood vessels. The liver was removed after perfusion, cut into small pieces by scissors and a scalpel, and digested with collagenase type IV (Sigma, St. Louis, MO, USA; 2.5 mg/ml) and DNase I (Promega, Madison, WI, USA; 1 mg/ml) for 15 min at 37°C. Subsequently, the digested tissues were dissociated into single-cell suspensions using a combination of C Tubes and a gentleMACS™ dissociator (Miltenyi, Bergisch Gladbach, Germany). The single-cell suspensions were filtered using a 70-μm-pore cell strainer, and subsequently, the cells were washed once with PBS (10% FBS). Mononuclear cells were collected from the 40/70% Percoll interphase after discontinuous Percoll gradient centrifugation. After washing with PBS, the number of total mononuclear cells was determined using 0.4% trypan blue (Welgene, Gyeongsan-si, Republic of Korea) and a hemocytometer before antibody staining (23).

### 2.6 Flow cytometry

The following monoclonal antibodies (mAbs) were obtained from BD Biosciences (San Jose, CA, USA): Fluorescein isothiocyanate (FITC)-conjugated anti-CD3ϵ (clone 145-2C11); FITC-, phycoerythrin (PE)- or PE/Cy7-conjugated anti-CD4 (clone RM4-5); FITC- or PE-conjugated anti-IL10 (clone JES5-16E3); PE- or PE/Cy7-conjugated anti-CD11b (clone M1/70); PE-conjugated anti-CD1d (clone 1B1); PE-conjugated anti-RORγt (clone Q31-378); PE-conjugated anti-NK1.1 (clone PK-136); PE-conjugated anti-TNFα (clone MP6-XT220); PE/Cy7-conjugated anti-CD69 (clone H1.2F3); PE/Cy7-conjugated anti-CD11c (clone HL3); PE/Cy7-conjugated anti-CD45 (clone 30-F11). The following mAbs from Thermo Fisher Scientific (Waltham, MA, USA) were used: FITC-, PE- or allophycocyanin (APC)-conjugated anti-CD19 (clone 1D3); FITC-conjugated anti-F4/80 (clone BM8); FITC-conjugated anti-NK1.1 (clone PK-136); PE-conjugated anti-FcϵRI (clone MAR-1); PE-conjugated anti-T-bet (clone 4B10); PE-conjugated anti-E4BP4 (clone S2M-E19); PE-conjugated anti-Nur77 (clone 12.14); PE-conjugated anti-PLZF (clone Mags.21F7); PE-conjugated anti-IL17A (clone eBio17B7); PE-conjugated anti-IL1β (clone NJTEN3); PE/Cy7-conjugated anti-CD23 (clone B3B4); PE/Cy7-conjugated anti-CD3ϵ (clone 145-2C11); FITC- or APC-conjugated anti-Ly6G (Gr-1) (clone 1A8-Ly6g); APC-conjugated anti-CD200R3 (clone Ba13). The following mAbs from BioLegend (San Diego, CA, USA) were used: FITC- or APC-conjugated anti-CD45 (clone 30-F11); PE-conjugated anti-CD21/CD35 (clone 7E9); PE/Cy7-conjugated anti-CD5 (clone 53-7.3); PE/Cy7-conjugated anti-CD1d (clone 1B1); PE/Cy7-conjugated anti-PLZF (clone 9E12); PE/Cy7-conjugated anti-CD19 (clone 6D5); APC-conjugated anti-CD3ϵ (clone 17A2). Flow cytometric data were acquired using a FACSCalibur flow cytometer (Becton Dickinson, San Jose, CA, USA) and analyzed using FlowJo software (version 8.7; Tree Star, Ashland, OR, USA). Cells were harvested and washed twice with cold 0.5% BSA-containing PBS (FACS buffer) to perform surface staining. The cells were incubated with anti-CD16/CD32 mAbs (clone 2.4G2) on ice for 10 min and stained with fluorescence-labeled mAbs to block the Fc receptor (24).

### 2.7 Intracellular cytokine/transcription factor staining

To perform intracellular cytokine/transcription factor staining, single-cell suspensions from the spleen were incubated for two hours at 37°C with intracellular protein transport inhibitor (brefeldin A, 10 μg/ml) in RPMI complete medium consisting of RPMI 1640 (Gibco BRL, Gaithersburg, MD, USA) medium supplemented with 10% FBS, 10 mM HEPES, 2 mM L-glutamine, 100 units/ml penicillin-streptomycin, and 5 mM 2-mercaptoethanol. The cells were stained for cell-specific surface markers, then fixed with 1% paraformaldehyde in PBS, washed with cold (4°C) FACS buffer, and permeabilized with 0.5% saponin in PBS for 10 min. Subsequently, the permeabilized cells were stained for 30 min at room temperature (RT) with the indicated anti-cytokine mAbs (FITC-conjugated anti-IL10, PE-conjugated anti-IL10, anti-TNFα, anti-IL17A, anti-IL1β, and FITC- or PE-conjugated rat IgG isotype control mAbs). Intracellular staining for transcription factors was performed using the Foxp3 staining buffer set kit (Thermo Fisher Scientific, Waltham, MA, USA) with the indicated mAbs (PE-conjugated anti-T-bet, anti-RORγt, anti-E4BP4, anti-PLZF, anti-Nur77 and PE/Cy7-conjugated anti-PLZF; PE or PE/Cy7-conjugated rat IgG isotype control mAbs). Samples (more than 5,000 cells) were acquired using a FACSCalibur, and the data were analyzed using the FlowJo software package (version 8.7; Tree Star, Ashland, OR, USA) (23, 24).

### 2.8 CD1d/α-GalCer dimer staining for iNKT cells

To stain iNKT cells specifically, mCD1d/Ig fusion proteins (CD1d dimer; mouse CD1d DimerX, BD Biosciences, San Jose, CA, USA) were incubated overnight at 37°C with a 40-fold molar excess of α-GalCer (in PBS containing 0.5% Tween 20). The staining cocktail was prepared by mixing α-GalCer-loaded mCD1d/Ig proteins with FITC-, PE- or APC-conjugated anti-mouse IgG1 Ab (clone A85-1, BD Biosciences, San Jose, CA, USA) at a 1:2 ratio of dimer to anti-mouse IgG1 Ab. Subsequently, the mixture was incubated for two hours at RT.

### 2.9 B cell enrichment by magnetically activated cell sorting

WT B6 mice were i.p. injected with α-GalCer (2 μg/mouse). Seven days later, splenocytes were harvested and stained with PE-conjugated anti-CD19 mAb. A single-cell suspension of splenocytes was prepared and resuspended in RPMI complete medium. CD19^+^ B cells from B6 mice were enriched by positive selection with anti-PE MACS and LD columns (Miltenyi Biotech, Bergisch Gladbach, Germany), following the manufacturer’s instructions. The MACS-purified CD19^+^ B cell population was >98% pure.

### 2.10 Analysis of liver sections

The livers were fixed in 4% paraformaldehyde, embedded in paraffin, and cut into 6 µm sections using a microtome (RM 2235, Leica, Wetzlar, Germany). The sections were stained with hematoxylin and eosin (H&E) to analyze histological changes.

### 2.11 Gene set enrichment analysis

GSEA was conducted using GSEA software (GSEA 4.2.1). Gene expression was analyzed in 14 patients using RNA-seq data. Significantly enriched gene sets were identified using p-value ≤0.05 and q-value ≤0.25 as a cutoff. Data were deposited in the NCBI’s Gene Expression Omnibus database (GEO GSE197775). GSEA from the Broad Institute (http://www.broad.mit.edu/gsea) was used to calculate the enrichment of genes in each set (25).

### 2.12 Statistical analysis

Statistical significance was determined using Excel (Microsoft, Redmond, WA, USA). Student’s *t*-test was performed for the comparison of two groups (* *p* < 0.05, ** *p* < 0.01, and *** *p* < 0.001 were considered significant in the Student’s *t*-test). Kaplan-Meier plots with a log-rank test was carried out using Statistics Kingdom (https://www.statskingdom.com/kaplan-meier.html) (accessed on 17 January 2023) (* *p* < 0.05, ** *p* < 0.01, and *** *p* < 0.001 were considered to be significant in the log-rank test).

## 3 Results

### 3.1 A single pre-administration of α-GalCer alters iNKT cell subsets and suppresses sepsis severity

**Figure 1:**
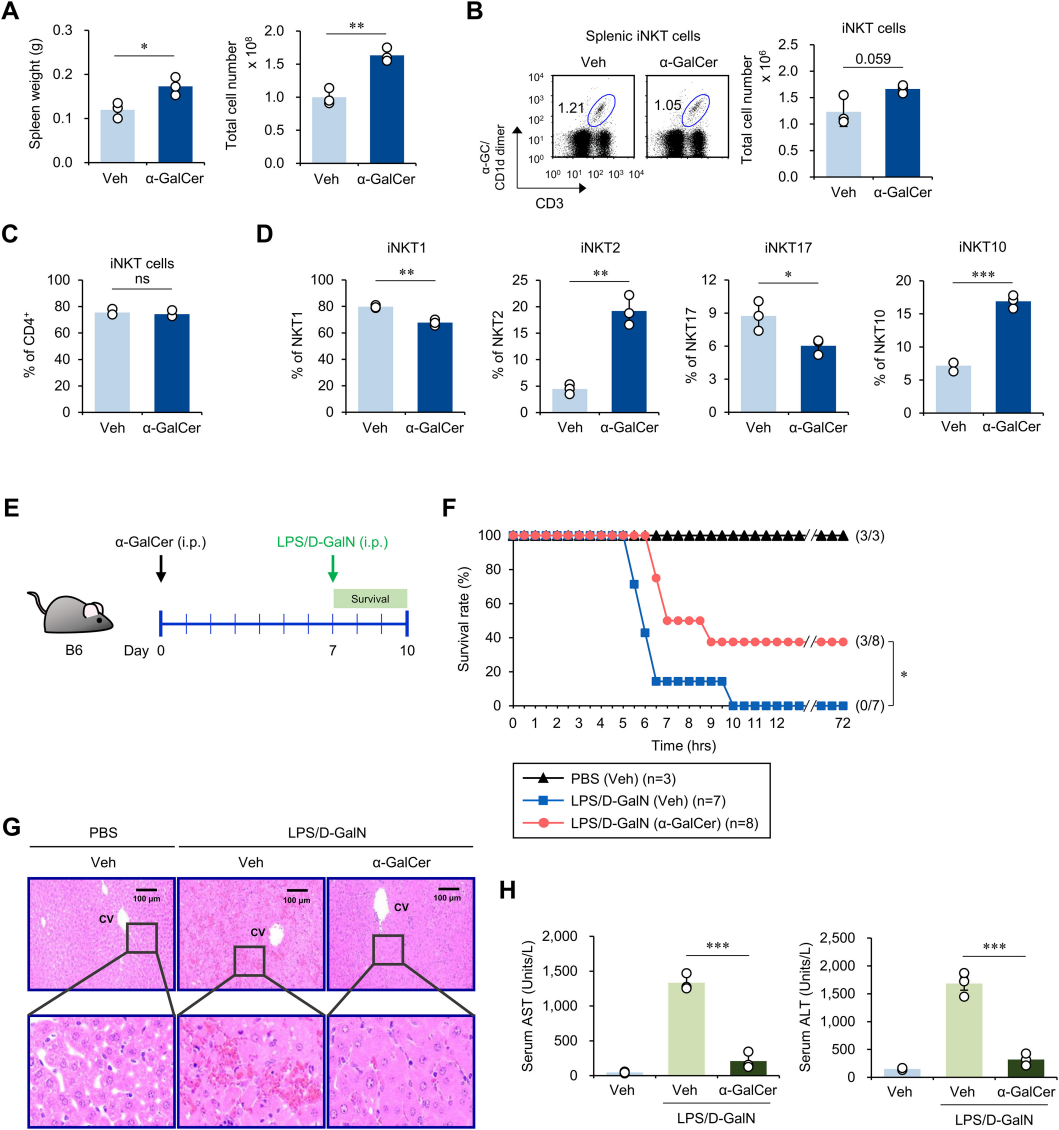
α-GalCer pretreatment alters iNKT cell subsets and attenuates sepsis severity. WT B6 mice were injected i.p. with α-GalCer (2 μg/mouse) and, seven days later, the spleens were harvested for the following analyses. **(A)** Weight and total cell number of spleens. **(B)** The frequency and cell number of splenic iNKT cells (α-GalCer/CD1d-dimer^+^CD3^+^). **(C)** The frequency of CD4^+^ iNKT cells in the spleen. **(D)** The relative frequencies of iNKT cell subsets (i.e., T-bet^+^ iNKT1, PLZF^+^ iNKT2, RORγt^+^ iNKT17, and E4BP4^+^ iNKT10 cells) were determined by flow cytometry. **(E)** Experimental outline: WT B6 mice were injected i.p. with α-GalCer (2 μg/mouse) and, seven days later, these mice were injected i.p. with LPS (2 µg/mouse) plus D-GalN (25 mg/mouse) for induction of sepsis. **(F)** Subsequently, these mice were monitored to evaluate their survival for three days. **(G, H)** H&E staining of liver sections (CV, central vein) **(G)** and serum levels of AST and ALT **(H)** were analyzed five hours after LPS/D-GalN injection. The mean values ± SD (*n* = 3 in **(A–D, H)**; per group in the experiment; Student’s *t*-test; *p < 0.05, **p < 0.01, and ***p < 0.001) are shown. The survival rate was analyzed by Kaplan-Meier plots with a log-rank test (*p < 0.05). One representative experiment of two experiments is shown. ns, not significant.

To investigate whether α-GalCer administration alters the iNKT cell subset distribution, WT B6 mice were injected i.p. with either α-GalCer or Veh and, seven days later, splenic iNKT cells were analyzed by flow cytometry. We found that spleen weights and total cell numbers were increased in α-GalCer-pretreated mice compared to Veh-injected controls (**Figure 1A**), indicating that α-GalCer pretreatment induces immune cell activation and proliferation. However, there were no significant changes in the total iNKT cell numbers and the prevalence of the CD4^+^ iNKT cell subset (**Figures 1B, C**). Next, we examined the profile of iNKT cell subsets based on the expression of subset-specific transcription factors for iNKT1 (T-bet^+^), iNKT2 (PLZF^+^), iNKT17 (RORγt^+^), and iNKT10 (E4BP4^+^) cells. We found that the frequencies of iNKT1 and iNKT17 cell subsets were decreased whereas those of the iNKT2 and iNKT10 cell subsets were increased in α-GalCer-pretreated mice (**Figure 1D**). Thus, our results revealed that a single administration of α-GalCer is sufficient to induce alterations in iNKT cell subsets in WT B6 mice. Because iNKT cells can modulate immune responses, we tested whether α-GalCer-induced alterations of iNKT cell subsets can affect the development of sepsis. To address this issue, we employed two different sepsis models, induced by LPS/D-GalN or α-GalCer/D-GalN in WT B6 mice. WT B6 mice pretreated with α-GalCer displayed a significantly higher survival rate than mice pretreated with Veh (**Figures 1E, F**; **Supplementary Figure S1A**). In addition, α-GalCer-pretreated mice showed significant decreases in liver immune cell infiltration and liver enzymes (aspartate transaminase [AST] and alanine transaminase [ALT]), which are common indicators of liver inflammation (**Figures 1G, H**; **Supplementary Figures S1B, C**). To support our findings in a more physiological manner, we employed the CS injection model, an alternative method to induce polymicrobial sepsis by i.p. injection of cecal contents (22). As expected, the survival rate of α-GalCer-pretreated mice was significantly increased in CS-induced sepsis (**Supplementary Figure S1D**). Therefore, we conclude that pre-administration of α-GalCer can attenuate sepsis severity.

### 3.2 α-GalCer pretreatment attenuates inflammatory responses in sepsis

Sepsis is accompanied by excessive inflammatory responses. For example, septic patients display increased serum levels of inflammatory cytokines (e.g., IL1, IL17A, and TNFα) and inflammatory immune cells (e.g., lymphocytes and neutrophils) than healthy individuals (26). To examine the immune cellularity in α-GalCer-pretreated mice, the spleen and liver were harvested and analyzed for tissue weights and total cell numbers. We found that α-GalCer pretreatment increased the splenic cell numbers in both untreated and LPS/D-GalN-treated mice (**Supplementary Figure S2A**). However, α-GalCer pretreatment dramatically reduced hepatic cell numbers in LPS/D-GalN-treated mice (**Supplementary Figure S2B**), suggesting inhibition of immune cell infiltration.

Next, we examined the absolute cell numbers of immune cells (i.e., neutrophils, T cells, NK cells, and NKT cells) known to participate in inflammatory immune responses caused by infection (27–29). Despite the immune-activating effects of α-GalCer (expansion of immune cells such as neutrophils), treatment with α-GalCer prior to LPS/D-GalN administration did not modify the total cell numbers of T cells, NK cells, and NKT cells in the spleen (**Supplementary Figure S2C**). However, interestingly, α-GalCer pretreatment significantly decreased the infiltration of immune cells to the liver following LPS/D-GalN injection (**Supplementary Figure S2D**). Collectively, our results suggest that the protective effects of α-GalCer pretreatment on LPS/D-GalN-induced sepsis are associated with reduced immune cell expansion and decreased infiltration of the liver.

**Figure 2:**
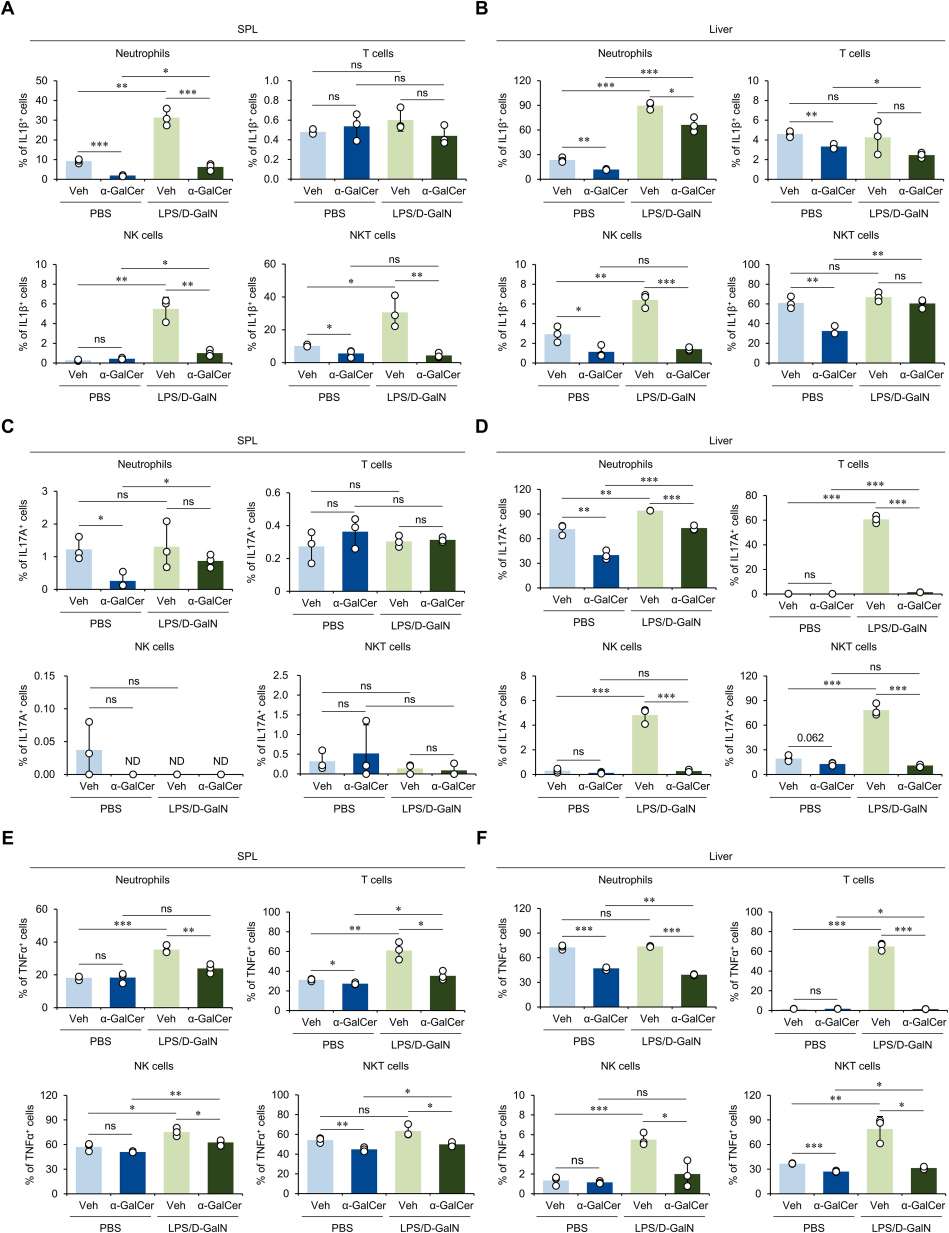
α-GalCer pretreatment attenuates the pro-inflammatory cytokine production by immune cells. WT B6 mice were injected i.p. with α-GalCer (2 μg/mouse) and, seven days later, these mice were injected i.p. with LPS (2 µg/mouse) plus D-GalN (25 mg/mouse) for induction of sepsis. Five hours later, the spleens and livers from these mice were harvested for the following analyses. **(A–F)** The frequencies of IL1β- **(A, B)**, IL17A- **(C, D)**, and TNFα-expressing populations **(E, F)** among neutrophils (Gr1^+^ CD11b^+^), T cells (CD3^+^ NK1.1^-^), NK cells (CD3^-^ NK1.1^+^), and NKT cells (CD3^+^ NK1.1^+^) in the spleen and liver. The mean values ± SD (*n* = 3 in **(A–F)**; per group in the experiment; Student’s *t*-test; *p < 0.05, **p < 0.01, and ***p < 0.001) are shown. One representative experiment of two experiments is shown. ns, not significant.

In addition, we examined whether α-GalCer pretreatment affects the production of pro-inflammatory cytokines involved in the development of various inflammatory diseases (2, 30). For this purpose, we analyzed the expression of inflammatory cytokines, including IL1β, IL17A, and TNFα, by neutrophils, T cells, NK cells, and NKT cells. We found that α-GalCer-pretreatment decreased IL1β expression by splenic neutrophils and NKT cells both with or without LPS/D-GalN administration (**Figure 2A**). Similar effects of α-GalCer pretreatment on IL1β expression were observed in the liver (**Figure 2B**). For IL17A, α-GalCer pretreatment affected its production by neutrophils in spleen in the absence but not presence of LPS/D-GalN administration (**Figure 2C**). However, α-GalCer pretreatment decreased IL17A production by immune cells, including neutrophils, T cells, NK cells, and NKT cells in the liver (**Figure 2D**). Furthermore, TNFα expression was also suppressed by α-GalCer pretreatment in the spleen (**Figure 2E**) and liver (**Figure 2F**) during LPS/D-GalN-induced sepsis. Thus, our results demonstrated that α-GalCer pretreatment reduces the production of inflammatory cytokines more prominently in the liver than in the spleen.

### 3.3 The protective effects of α-GalCer pretreatment do not depend on IL4-STAT6 signaling

**Figure 3:**
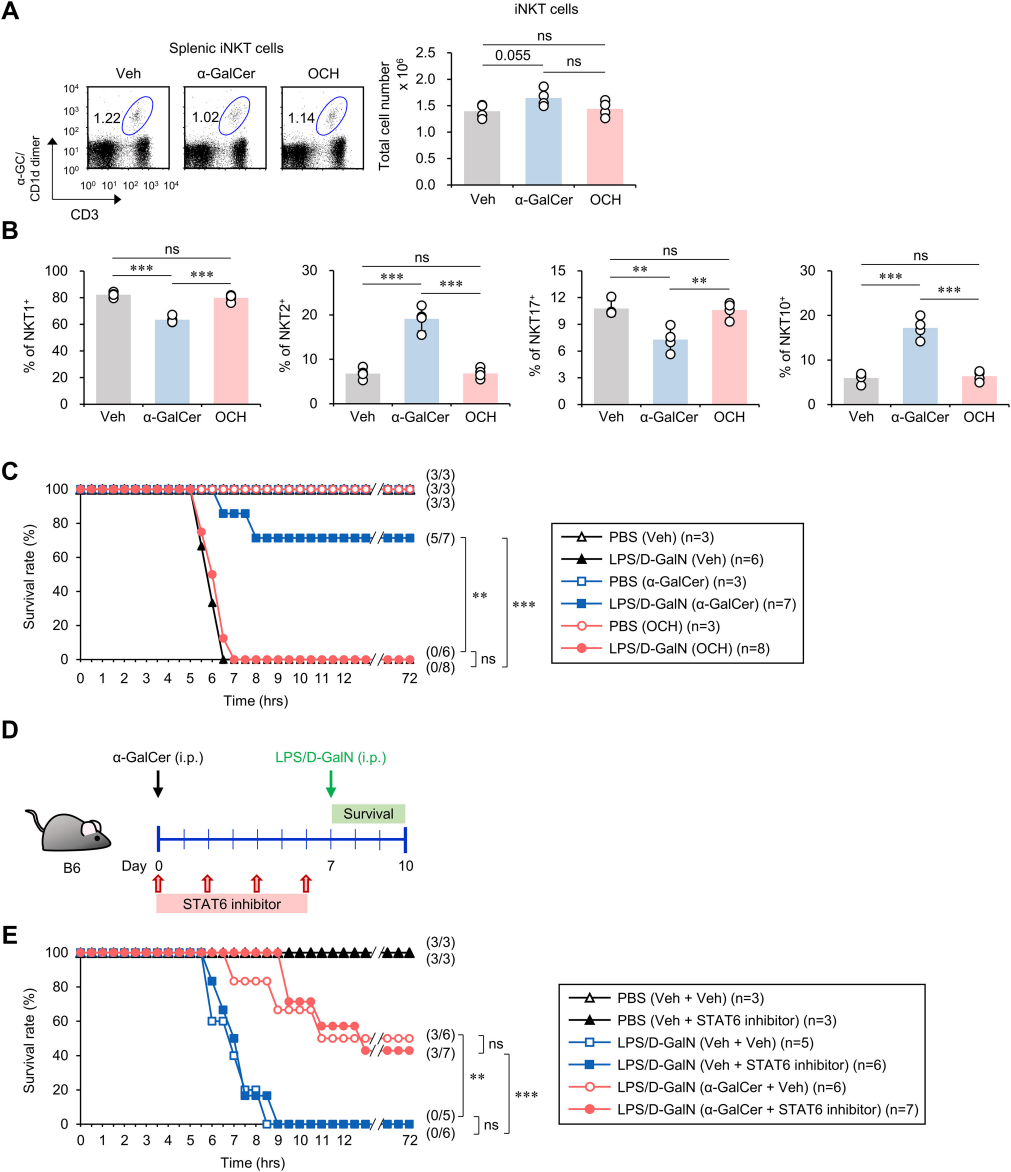
The preventive effects of α-GalCer pretreatment on sepsis do not correlate with the IL4-STAT6 signaling pathway. WT B6 mice were injected i.p. with α-GalCer (2 μg/mouse) or OCH (2 μg/mouse) and, seven days later, the spleens were harvested. **(A)** The frequency and absolute cell number of splenic iNKT cells were determined by flow cytometry. **(B)** Flow cytometric analysis for the expression of transcription factors (T-bet, PLZF, RORγt, and E4BP4) by splenic iNKT cells. **(C)** WT B6 mice were injected i.p. with α-GalCer (2 μg/mouse) or OCH (2 μg/mouse) and, seven days later, mice were injected i.p. with LPS (2 µg/mouse) plus D-GalN (25 mg/mouse) for induction of sepsis. Subsequently, these mice were monitored to evaluate their survival for three days after LPS/D-GalN injection. **(D)** Experimental outline: WT B6 mice were injected i.p. with α-GalCer (2 μg/mouse) on day 0 and, seven days later, mice were injected i.p. with LPS (2 µg/mouse) plus D-GalN (25 mg/mouse) for induction of sepsis. To evaluate the effect of IL4 signaling on α-GalCer-mediated attenuation of sepsis, WT B6 mice were injected i.p. four times with a STAT6 inhibitor (AS1517499, 10mg/kg) every other day starting from day 0. **(E)** Subsequently, these mice were monitored to evaluate their survival for three days after LPS/D-GalN injection. The mean values ± SD (n = 4 in **(A, B)**; per group in the experiment; Student’s *t*-test; **p < 0.01 and ***p < 0.001) are shown. The survival rate was analyzed by Kaplan-Meier plots with a log-rank test (**p < 0.01 and ***p < 0.001). One representative experiment of two experiments is shown. ns, not significant.

It has been previously reported that OCH, an analog of α-GalCer, can activate and polarize iNKT cells towards an IL4-biased type 2 cytokine production phenotype (31, 32). Since α-GalCer pretreatment increased the IL4-producing iNKT2 cell subset (**Figure 1D**), we reasoned that OCH pretreatment may confer similar or more enhanced protective effects against sepsis than α-GalCer. First, we confirmed that OCH treatment activated iNKT cells (**Supplementary Figure S3A**). Next, we confirmed the effects of OCH pretreatment on iNKT cells. Similar to α-GalCer treatment, OCH treatment did not affect the total cell numbers of iNKT cells (**Figure 3A**). However, unlike α-GalCer treatment, OCH treatment also did not affect the iNKT cell subset composition (**Figure 3B**). To investigate the impact of OCH on sepsis, we treated WT B6 mice with α-GalCer or OCH one week prior to induction of sepsis by LPS/D-GalN or α-GalCer/D-GalN injection. Unexpectedly, we found a dramatic difference in the mortality rate between the α-GalCer and OCH treatment groups (**Figure 3C**; **Supplementary Figure S3B**), indicating the absence of prophylactic effects of OCH on sepsis development.

To investigate the effect of α-GalCer on IL4 production, we analyzed splenic and hepatic IL4-producing CD4^+^ T cells in α-GalCer-pretreated mice. We found reduced splenic IL4^+^ cells in α-GalCer-treated compared to Veh-treated mice, while no significant difference was observed for hepatic IL4^+^ cells (**Supplementary Figure S4A**). We found reduced frequency of IL4-producing CD4^+^ T cells in the spleen, but no difference in the liver (**Supplementary Figure S4B**). In addition, since basophil-derived IL4 can promote tissue repair following liver damage induced by bacterial infections (33), we examined whether α-GalCer pretreatment induces IL4 expression by basophils. We found that neither the frequency nor the number of basophils producing IL4 in the spleen and liver were increased (**Supplementary Figures S4C, D**), implying that IL4-producing immune cells such as CD4^+^ T cells and basophils are not essential for mediating the protective effects of α-GalCer pretreatment against sepsis.

Next, to confirm that the IL4 signaling pathway does not play a substantial role in the protection conferred by α-GalCer against sepsis, we employed AS1517499, an inhibitor of the STAT6 transcription factor that is critical for IL4 signaling (34). Alpha-GalCer-treated WT B6 mice were injected 4 times with AS1517499 every other day prior to sepsis induction by LPS/D-GalN (**Figure 3D**) or α-GalCer/D-GalN (**Supplementary Figure S3C**). We found that the STAT6 inhibitor did not cause any significant differences in mortality rates in α-GalCer-treated mice compared to PBS-injected controls (**Figure 3E**; **Supplementary Figure S3D**), suggesting that α-GalCer pretreatment confers its protective effects independently of IL4-STAT6 signaling.

### 3.4 The anti-inflammatory effects of α-GalCer pretreatment on septic shock correlate with the expansion of IL10-producing immune cells

**Figure 4:**
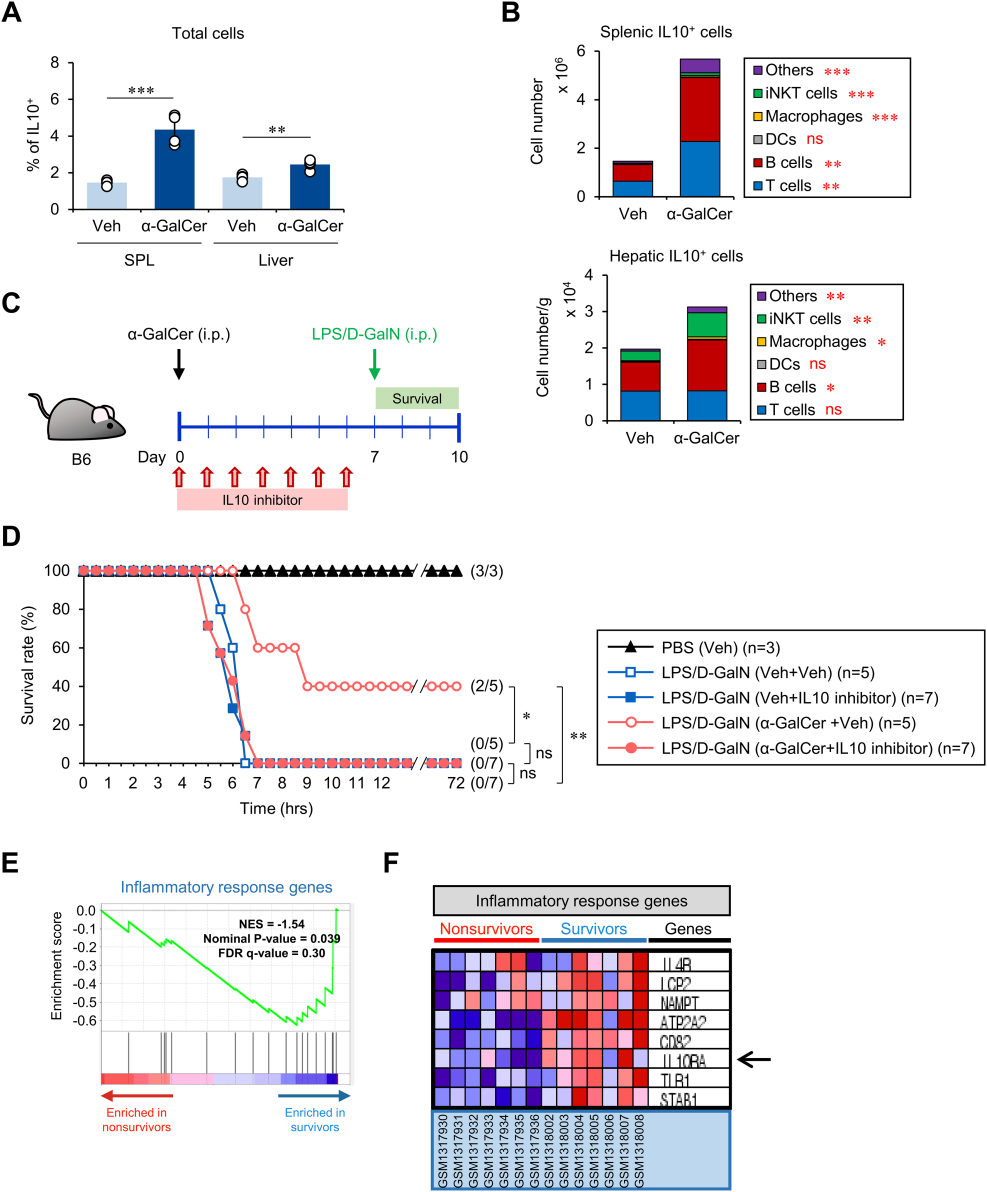
The preventive effects of α-GalCer pretreatment correlate with the expansion of IL10^+^ immune cells. WT B6 mice were injected i.p. with α-GalCer (2 μg/mouse) and, seven days later, the spleens and livers were harvested for the following analyses. **(A)** The frequency of IL10^+^ cells in the spleen and liver. **(B)** The absolute cell numbers of IL10-producing cells (i.e., T cells, B cells, DCs, macrophages, iNKT cells, and others) in the spleen and liver were determined by flow cytometry. **(C)** Experimental outline: WT B6 mice were injected i.p. with α-GalCer (2 μg/mouse) on day 0. Subsequently, these mice were injected i.p. with an IL10 inhibitor (AS101, 10 μg/mouse) daily for one week starting from day 0. On day 7, these mice were injected i.p. with LPS (2 µg/mouse) plus D-GalN (25 mg/mouse) for induction of sepsis. **(D)** Subsequently, these mice were monitored to evaluate their survival for three days after LPS/D-GalN injection. **(E, F)** Comparison of transcriptional profiles of inflammatory response genes in PBMCs of either non-survivors (red) or survivors (blue) of sepsis in human subjects by relevant gene set enrichment plots from GSEA (NES, normalized enrichment score; FDR, false discovery rate). The mean values ± SD (n = 4 in **(A, B)**; per group in the experiment; Student’s *t*-test; *p < 0.05, **p < 0.01, and ***p < 0.001) are shown. The survival rate was analyzed by Kaplan-Meier plots with a log-rank test (*p < 0.05 and **p < 0.01). One representative experiment of two experiments is shown. ns, not significant.

To elucidate the mechanism by which α-GalCer pretreatment protects against sepsis, we considered our finding that α-GalCer pretreatment augments the iNKT10 cell subset (**Figure 1D**), as prior studies have demonstrated protective effects of the anti-inflammatory cytokine IL10 in sepsis (35, 36). We found that α-GalCer pretreatment increased the frequency of IL10-producing immune cells in the spleen and liver (**Figure 4A**). In addition, we tested whether α-GalCer pretreatment alters the composition of IL10-producing immune cells (i.e., T cells, B cells, DCs, macrophages, iNKT cells, and others) in the spleen and liver. We found that α-GalCer pretreatment significantly expanded the numbers of IL10-producing iNKT cells, B cells, and macrophages in the spleen and liver (**Figure 4B**). Next, we examined whether α-GalCer-induced IL10 can affect the outcome of septic shock by employing an IL10 inhibitor (AS101) (**Figure 4C**; **Supplementary Figure S5A**). While the survival rate of B6 mice treated with α-GalCer was significantly improved, administration of the IL10 inhibitor diminished the protective effects of α-GalCer against septic shock (**Figure 4D**). Moreover, we found that IL10 inhibition more profoundly affected the severity of iNKT-mediated sepsis (**Supplementary Figure S5B**). These results provide strong support for the critical role of IL10 in mediating the capacity of α-GalCer to protect against sepsis.

To provide evidence that IL10-mediated immune responses correlate with protection against sepsis pathogenesis in human, we performed GSEA on PBMCs of groups of sepsis survivors and non-survivors. We found that survivors downregulated inflammatory response genes (**Figure 4E**) but upregulated the IL10RA gene (**Figure 4F**), implying that α-GalCer-induced, IL10-associated phenotypic changes could be utilized as a therapeutic approach in humans.

### 3.5 α-GalCer pretreatment expands Breg cells that partially contribute to sepsis protection upon adoptive transfer

**Figure 5:**
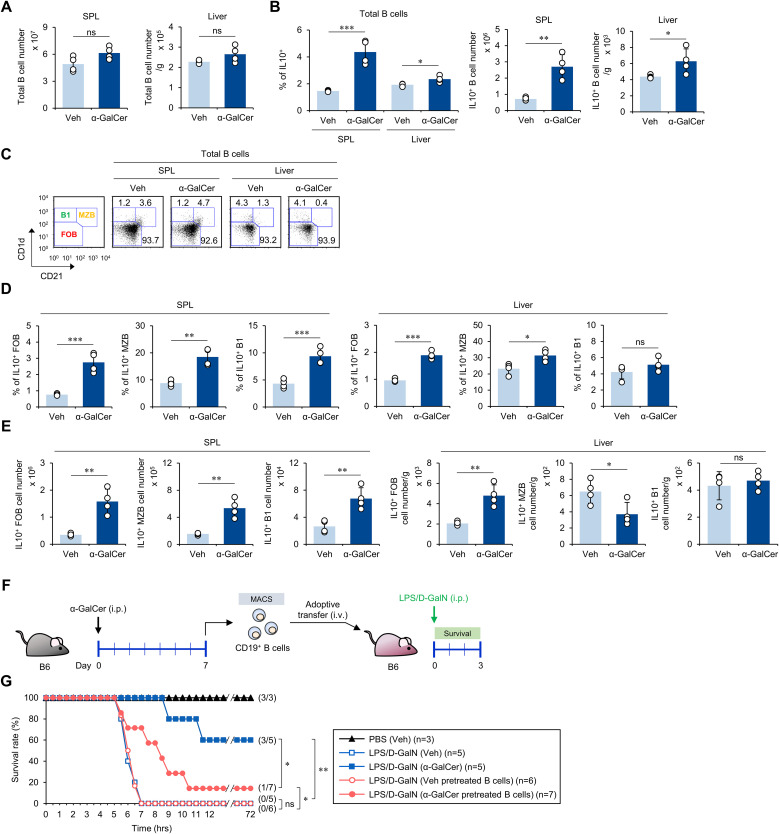
α-GalCer pretreatment expands IL10-producing B cells. WT B6 mice were injected i.p. with α-GalCer (2 μg/mouse) and, seven days later, the spleens and livers were harvested for the following analyses. **(A, B)** The total CD19^+^ B cell numbers **(A)** and the frequency and absolute cell numbers **(B)** of IL10-producing B cells in the spleen and liver. **(C)** The frequencies of B cell subsets (FOB, MZB, and B1 cells) in the spleen and liver. **(D, E)** The frequencies **(D)** and absolute cell numbers **(E)** of IL10-producing B cell subsets (FOB, MZB, and B1 cells) in the spleen and liver. **(F)** Experimental outline: WT B6 mice were injected i.p. with α-GalCer (2 μg/mouse) and, seven days later, splenic CD19^+^ B cells were isolated by the MACS system. Subsequently, MACS-purified CD19^+^ B cells (5 × 10^6^ cells/mouse) were adoptively transferred to recipient mice. The recipient mice were injected i.p. with LPS (2 µg/mouse) plus D-GalN (25 mg/mouse) for induction of sepsis. **(G)** Subsequently, these mice were monitored to evaluate their survival for 3 days after LPS/D-GalN. The mean values ± SD (n = 4 in **(A, B, D, E)**; per group in the experiment; Student’s *t*-test; *p < 0.05, **p < 0.01, and ***p < 0.001) are shown. The survival rate was analyzed by Kaplan-Meier plots with a log-rank test (*p < 0.05 and **p < 0.01). One representative experiment of two experiments is shown. ns, not significant.

It has been reported that IL10-producing Breg cells can suppress exacerbated inflammatory immune responses (13). Moreover, iNKT cells can assist in the expansion of IL10-producing Breg cells (37). α-GalCer treatment increased both the frequency and absolute cell number of IL10-producing B cells in the spleen and liver, although no significant changes in total B cell numbers were observed (**Figures 5A, B**). Next, to identify the B cell subset producing IL10 upon α-GalCer pre-stimulation, we analyzed B cell subsets based on the expression of CD1d and CD21 molecules, to distinguish between follicular B (FOB) cells, marginal zone B (MZB) cells, and B1 cells (**Figure 5C**). Although α-GalCer pretreatment did not influence the cell number of FOB and B1a cells, intriguingly, the number of MZB cells increased in the spleen but decreased in the liver (**Supplementary Figures S6A, B**). Although there were no significant alterations in cell numbers, α-GalCer pretreatment dramatically increased the frequency and absolute cell numbers of IL10-producing B cells, except for hepatic MZB and B1 cell populations (**Figures 5D, E**). Thus, our results demonstrate that α-GalCer pretreatment induces IL10-producing Breg cell populations, strongly suggesting that IL10^+^ FOB cells might be prominent contributors to IL10 production.

Since it has been reported that B cell-derived IL10 is critical in resolving LPS-induced acute lung injury (38), we next investigated whether Breg cells induced by α-GalCer can protect against sepsis. We isolated splenic CD19^+^ B cells from WT B6 mice pretreated with either Veh or α-GalCer, adoptively transferred these B cells to WT B6 mice, and induced sepsis in the recipient animals by injection with LPS/D-GalN (**Figure 5F**). We found that adoptive transfer of B cells from α-GalCer-pretreated mice slowed the progression of sepsis development compared with B cells from Veh-pretreated mice. This protection was only partial (**Figure 5G**), suggesting that α-GalCer-mediated expansion of IL10-producing B cells partially contributes to alleviating septic shock.

## 4 Discussion

In summary, the present study demonstrates that α-GalCer pretreatment can regulate inflammatory immune responses such as septic shock by shifting iNKT and B cells towards anti-inflammatory phenotypes, particularly expression of the anti-inflammatory cytokine IL10.

It has been reported that IL10 levels are elevated in the blood of patients with sepsis (39). Such IL10 increases might reflect mechanisms in patients with systemic inflammatory response syndrome that counteract organ failure (35). It has been reported that IL10 inhibits the differentiation of naive CD4 T cells into effector Th cells by inhibiting IL12/IL4 production and DC function, thereby modulating both Th1-type and Th2-type responses (40). Our results in **Supplementary Figure S4B** imply that IL10 (from iNKT10 cells and Breg cells) produced by α-GalCer-pretreatment (**Supplementary Figures S7A, B**) may contribute to downregulating IL4-mediated immune responses.

A recent study demonstrated that IL10^+^ macrophages expanded during LPS-induced sepsis promote homeostasis and survival (41). Consistent with this report, our results show that α-GalCer pretreatment increases IL10-producing macrophages in the liver (**Figure 4B**), which might be associated with counteracting inflammatory immune responses. Thus, it will be interesting to further investigate the phenotype of IL10^+^ macrophages induced by α-GalCer pretreatment.

Inadequate migration of neutrophils to injured target organs contributes to the systemic inflammatory response associated with high sepsis mortality rates (27). In addition, severe sepsis is associated with a decrease in circulating neutrophils due to increased apoptosis (42). Interestingly, we found that α-GalCer pretreatment increased splenic neutrophil numbers in LPS/D-GalN-injected mice, and opposite findings were observed in the liver (**Supplementary Figures S2C, D**), suggesting that α-GalCer pretreatment may affect neutrophil apoptosis and migration. Furthermore, our data in **Figure 2** demonstrated that α-GalCer treatment suppresses production of IL1β and TNFα by N1-type neutrophils (43). In addition, it has been reported that anti-inflammatory N2-type neutrophils display increased viability as compared with pro-inflammatory N1-type neutrophils (44). Together, our results suggest that α-GalCer pretreatment can alter the phenotype of neutrophils from a pathogenic N1-type towards a protective N2-type.

Our data demonstrated that α-GalCer pretreatment reduces the prevalence of splenic iNKT17 cells (**Figure 1D**). Furthermore, IL17A production by hepatic NKT cells was significantly decreased in α-GalCer-pretreated mice during LPS/D-GalN-induced sepsis (**Figure 2D**). Since IL17 signaling is crucial in recruiting neutrophils (45), it is likely that α-GalCer pretreatment modulates IL17A production by iNKT cells, which, in turn, overcomes the defect in liver neutrophil recruitment. This possibility will be explored in future studies.

Although much progress has been made in the medical field, postoperative sepsis remains a significant contributor to in-hospital mortality (46, 47). From this point of view, our results suggest a novel approach to preventing postoperative sepsis, by promoting anti-inflammatory conditions prior to surgery and potential exposures to microbial infection. This scenario was tested in **Supplementary Figure S1D** by employing a CS-induced sepsis model mimicking post-operative sepsis. Moreover, from GSEA of patient PBMCs of both survivor and non-survivor groups (**Figures 4E, F**), we found that the survivor group downregulates inflammatory response genes and upregulates IL10 responsiveness by increasing IL10RA gene expression (**Figure 4F**). Therefore, our study suggests α-GalCer treatment as a prophylactic option to prevent sepsis in humans.

## 5 Conclusions

Here, we demonstrated that α-GalCer pretreatment polarizes iNKT cells from a pro-inflammatory phenotype (i.e., iNKT1 cells and iNKT17 cells) towards an anti-inflammatory phenotype (i.e., iNKT2 cells and iNKT10 cells) that attenuates sepsis severity. Such preventive effects of α-GalCer pretreatment on sepsis development were attributed to reduced pro-inflammatory cytokine production (i.e., TNFα and IL1β) and immune cell infiltration to the liver. Our results identify iNKT cells as critical players in controlling type 1 inflammatory responses during sepsis, with potential prophylactic implications.

## Data Availability

The raw data supporting the conclusions of this article will be made available by the authors, without undue reservation.
