# Supplementary material for: The iNKT cell ligand α-GalCer prevents murine septic shock by inducing IL10-producing iNKT and B cells
Source: Front Immunol. 2024 Sep 17;15:1457690. doi: 10.3389/fimmu.2024.1457690 (PMC11442275; doi:10.3389/fimmu.2024.1457690)
Supplement: Supplementary file 1 [file DataSheet1.docx]

**Supplementary Materials to:**

**The iNKT cell ligand α-GalCer prevents murine septic shock by inducing IL10-producing iNKT and B cells**

**This document includes:**

**-Supplementary Fig. 1-7**


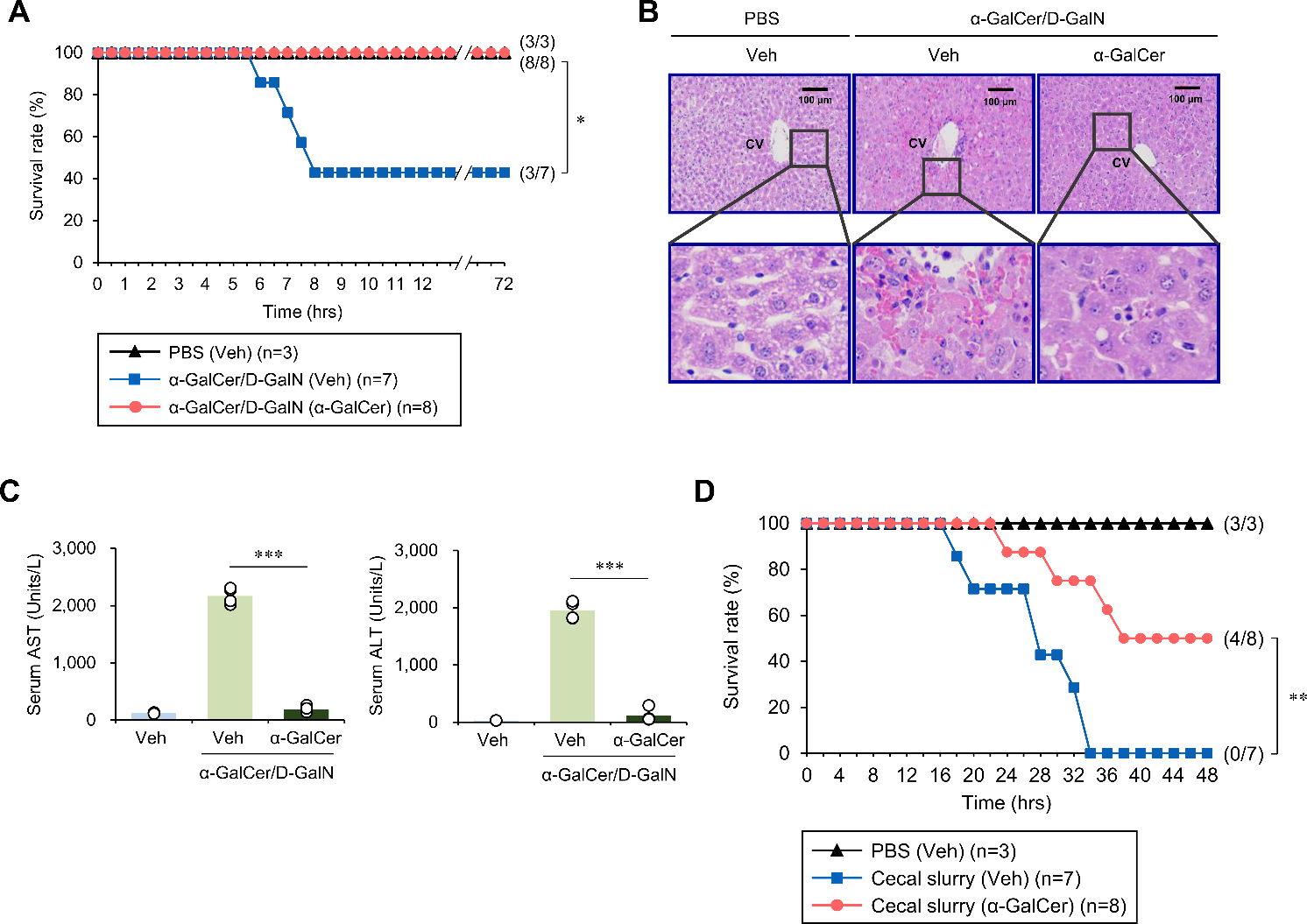


**Supplementary Figure 1. α-GalCer pretreatment protects mice from sepsis in two different sepsis models.**

In addition to the LPS/D-GalN-induced sepsis model, we evaluated the preventive effect of α-GalCer pretreatment on sepsis using two additional sepsis models (α-GalCer/D-GalN- and CS-induced sepsis models). WT B6 mice were injected i.p. with α-GalCer (2 μg/mouse) and, seven days later, mice were injected i.p. with α-GalCer (2 µg/mouse) plus D-GalN (10 mg/mouse). **(A)** Subsequently, these mice were monitored to evaluate survival rates for the following three days. **(B, C)** Liver sections were stained with H&E **(B)** and serum AST and ALT levels **(C)** were analyzed 5 hours after α-GalCer/D-GalN injection (CV, central vein). **(D)** WT B6 mice were injected i.p. with either Veh or α-GalCer (2 µg/mouse) and, seven days later, mice were injected i.p. with CS (1.3 mg feces/g body weight) for induction of sepsis. Subsequently, these mice were monitored to evaluate survival rates for the following two days. The mean values ± SD (n = 4 in C; per group in the experiment; Student’s *t*-test; *** *p* < 0.001) are shown. The survival rate was analyzed by Kaplan-Meier plots with a log-rank test (* *p* < 0.05 and ** *p* < 0.01). One representative experiment of two experiments is shown.


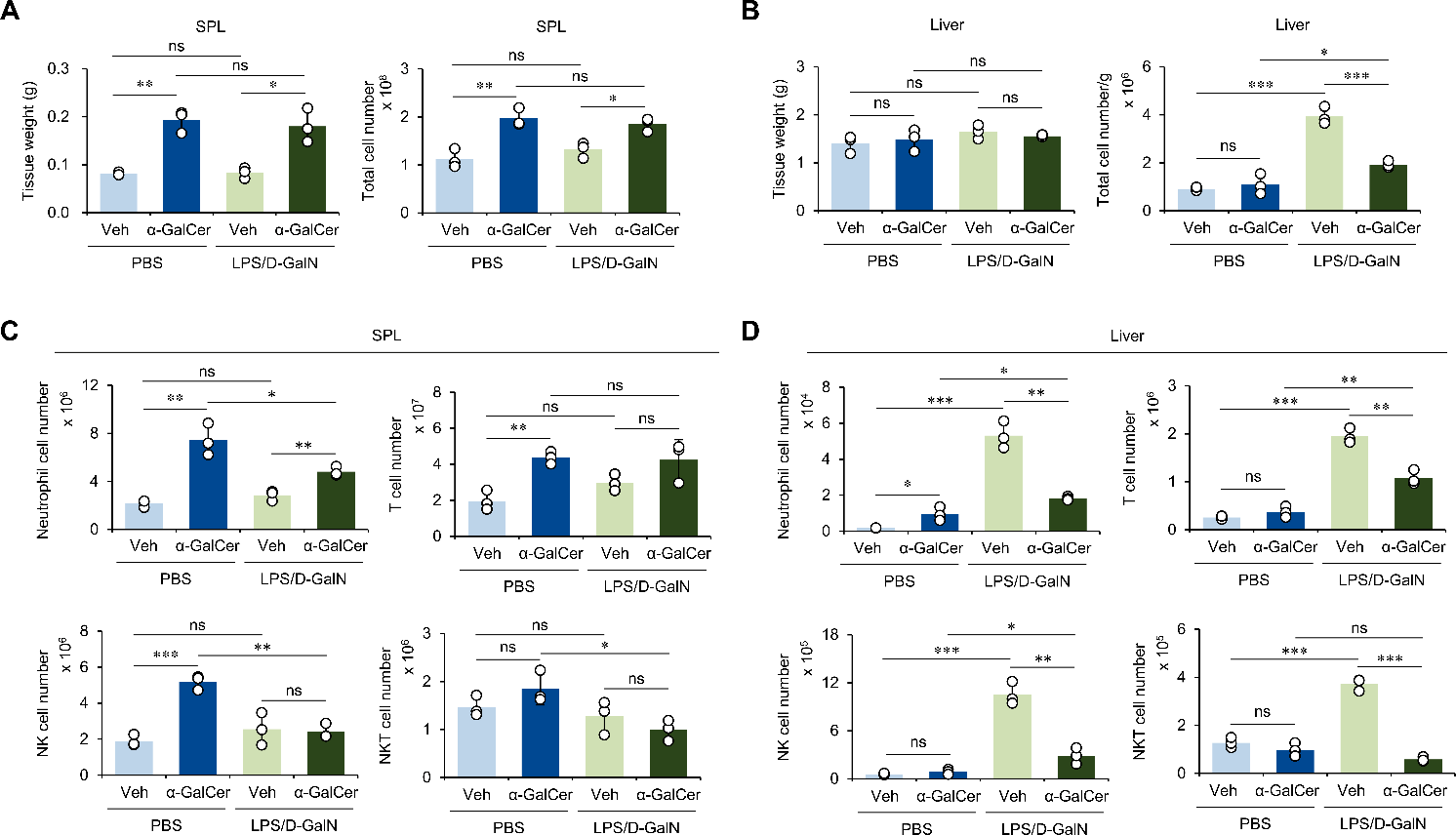


**Supplementary Figure 2. α-GalCer pretreatment reduces the infiltration of immune cells to the liver during sepsis.**

WT B6 mice were injected i.p. with α-GalCer (2 μg/mouse) and, seven days later, these mice were injected i.p. with LPS (2 µg/mouse) plus D-GalN (25 mg/mouse) for induction of sepsis. Five hours postinjection of LPS/D-GalN, the spleens and livers from these mice were harvested for the following analyses. **(A, B)** Tissue weight and leukocyte numbers in the spleen **(A)** and liver **(B)**. **(C, D)** The number of neutrophils (Gr1⁺ CD11b⁺), T cells (CD3⁺ NK1.1⁻), NK cells (CD3⁻ NK1.1⁺), and NKT cells (CD3⁺ NK1.1⁺) in the spleen **(C)** and liver **(D)**. The mean values ± SD (*n* = 3 in A-D; per group in the experiment; Student’s *t*-test; * *p* < 0.05, ** *p* < 0.01, and *** *p* < 0.001) are shown. One representative experiment of two experiments is shown. ns, not significant.

**
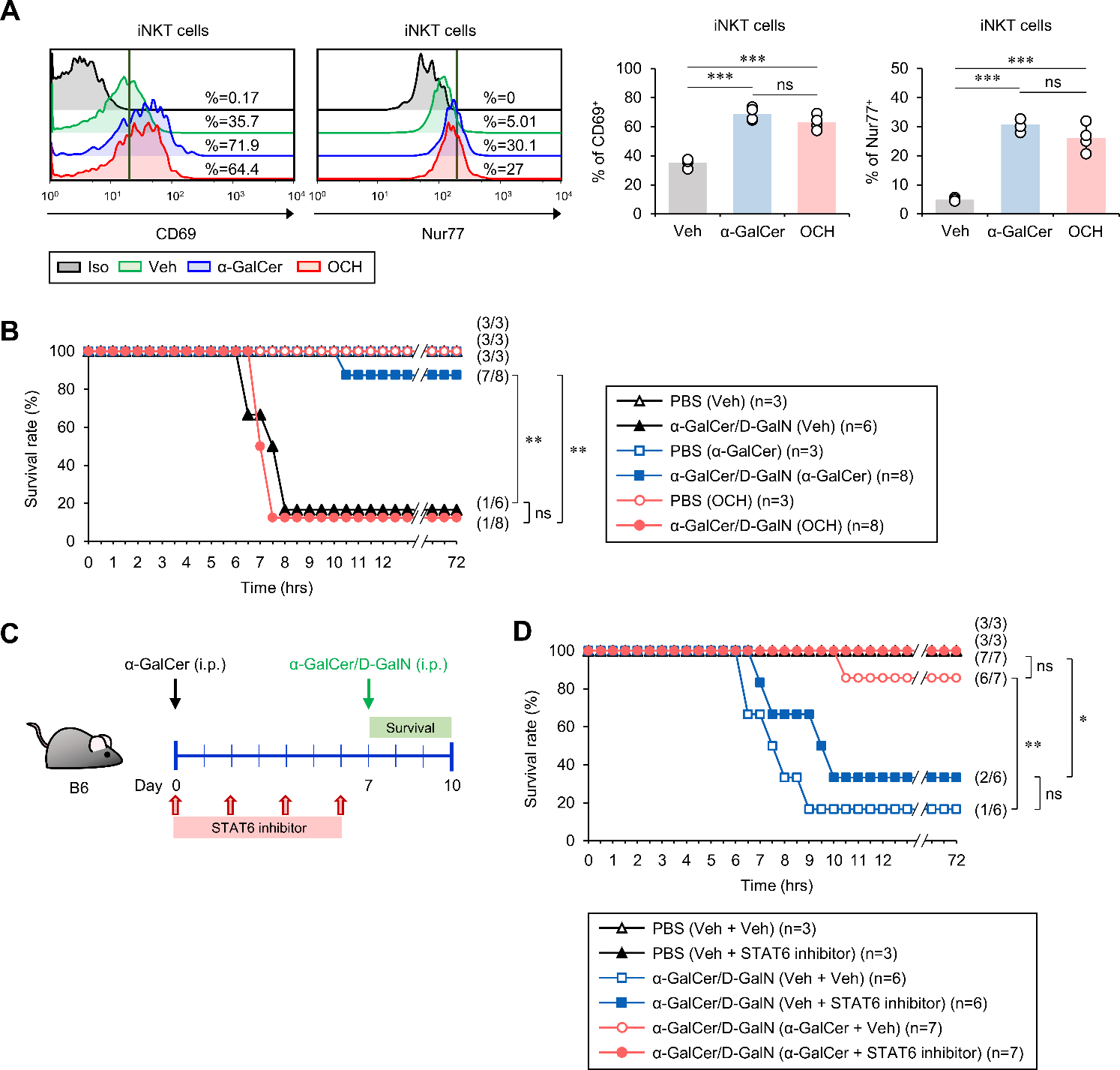
**

**Supplementary Figure 3. IL4-STAT6 signaling does not contribute to the protective effects of α-GalCer pretreatment on α-GalCer/D-GalN-induced sepsis.**

**(A)** The expression of CD69 and Nur77 by iNKT cells (α-GalCer/CD1d-dimer⁺ CD3⁺). **(B)** WT B6 mice were injected i.p. with α-GalCer (2 μg/mouse) or OCH (2 μg/mouse) and, seven days later, mice were injected i.p. with α-GalCer (2 µg/mouse) plus D-GalN (10 mg/mouse). Subsequently, these mice were monitored to evaluate survival rates for the following three days. **(C)** Experimental outline: WT B6 mice were injected i.p. with α-GalCer (2 μg/mouse) on day 0 and, seven days later, mice were injected i.p. with α-GalCer (2 µg/mouse) plus D-GalN (10 mg/mouse) for induction of sepsis. To evaluate the effect of IL4 signaling on α-GalCer-pretreatment-mediated sepsis attenuation, WT B6 mice were injected i.p. four times with a STAT6 inhibitor (AS1517499, 10 mg/kg) every other day starting from day 0 before sepsis induction. **(D)** Subsequently, these mice were monitored to evaluate their survival for three days after α-GalCer/D-GalN. The mean values ± SD (n = 4 in A; per group in the experiment; Student’s t-test; *** *p* < 0.001) are shown. The survival rate was analyzed by Kaplan-Meier plots with a log-rank test (* *p* < 0.05 and ** *p* < 0.01). One representative experiment of two experiments is shown. ns, not significant.

**
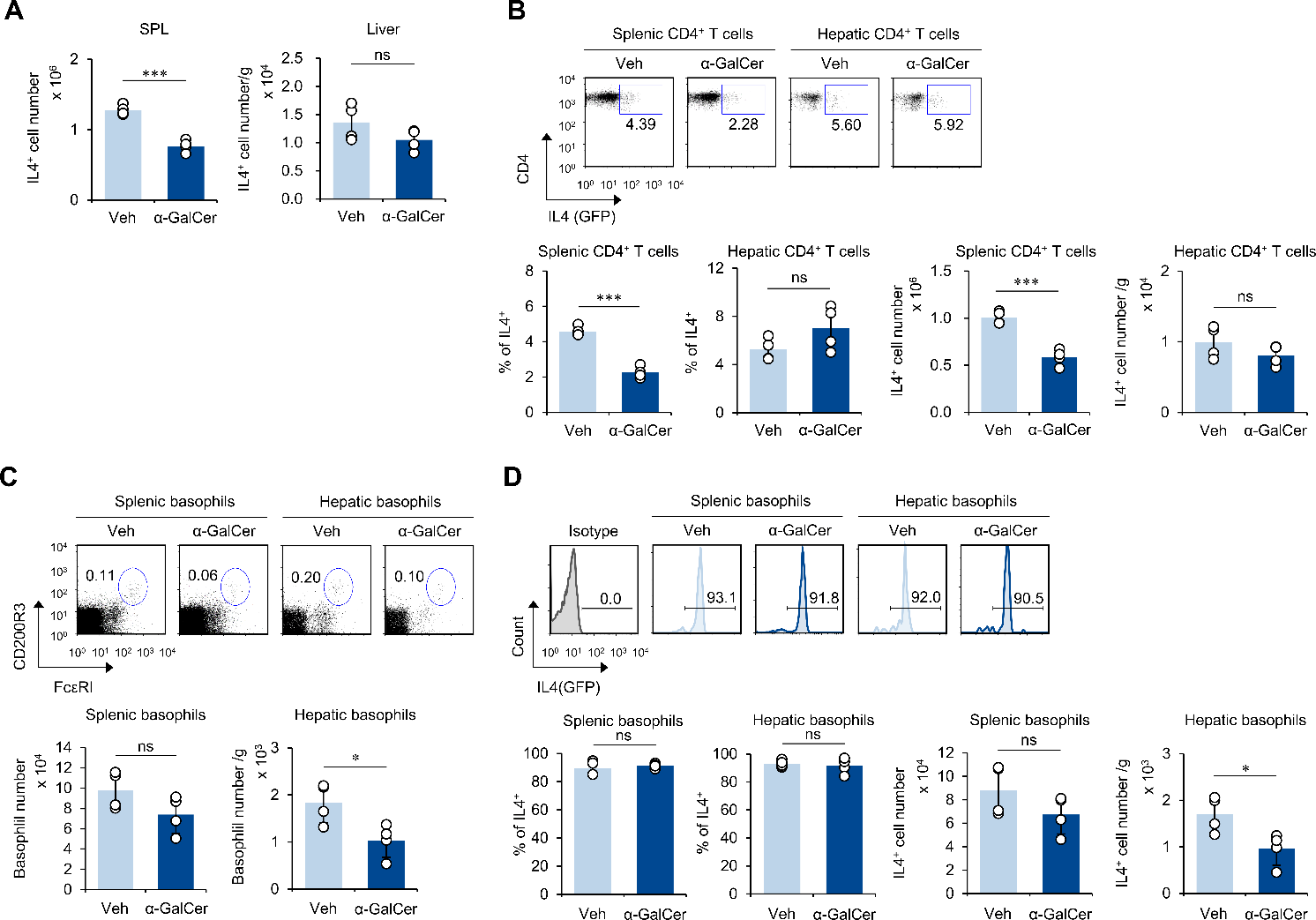
**

**Supplementary Figure 4. α-GalCer pretreatment does not increase the frequency of IL4(GFP)^+^ CD4⁺ T cells and basophils.**

IL4-GFP cytokine reporter (4Get) B6 mice were injected i.p. with α-GalCer (2 μg/mouse) and, seven days later, the spleen and liver were harvested for flow cytometric analysis. **(A)** IL4 (GFP)⁺ cell numbers in spleen and liver. **(B-D)** The frequency and cell numbers of splenic and hepatic IL4⁺ CD4⁺ T cells (CD3⁺ CD4⁺) **(B)** and IL4⁺ basophils (CD200R3⁺ FcεRⅠ⁺) **(C, D)** were determined by flow cytometry. The mean values ± SD (n = 4 in A-D; per group in the experiment; Student’s t-test; (* *p* < 0.05 and *** *p* < 0.001) are shown. One representative experiment of two experiments is shown. ns, not significant.

**
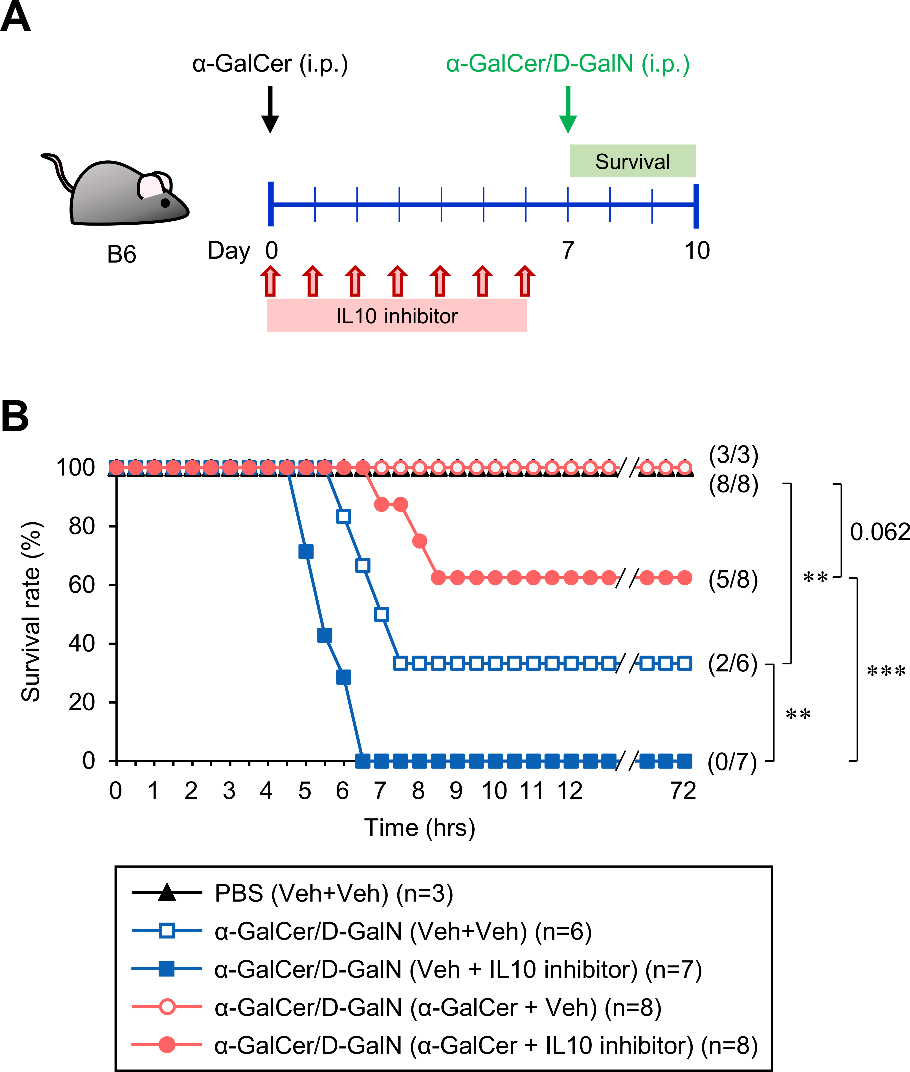
**

**Supplementary Figure 5. IL10 plays a crucial role in α-GalCer-pretreatment-mediated attenuation of α-GalCer/D-GalN-induced sepsis.**

**(A)** Experimental outline: WT B6 mice were injected i.p. with α-GalCer (2 μg/mouse) on day 0. Subsequently, these mice were i.p. injected with an IL10 inhibitor (AS101, 10 μg/mouse) daily for one week starting from day 0. On day 7, these mice were injected i.p. with α-GalCer (2 µg/mouse) plus D-GalN (10 mg/mouse) for induction of sepsis. **(B)** Subsequently, these mice were monitored to evaluate their survival for three days after α-GalCer/D-GalN. The survival rate was analyzed by Kaplan-Meier plots with a log-rank test (** < 0.01 and *** *p* < 0.001). One representative experiment of two experiments is shown.

**
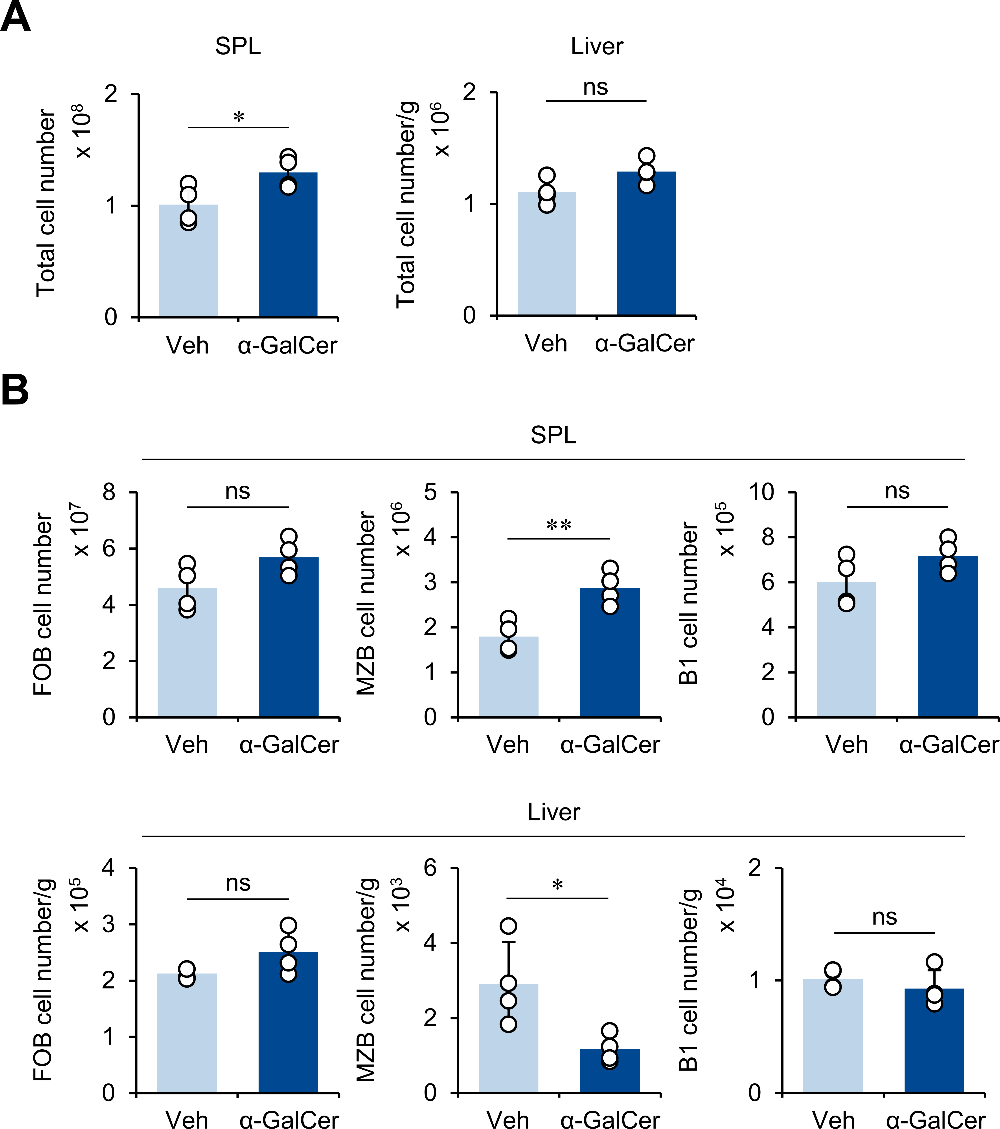
**

**Supplementary Figure 6. Splenic and hepatic B cell subsets after α-GalCer pretreatment.**

WT B6 mice were injected i.p. with α-GalCer (2 μg/mouse) and, seven days later, the spleen and liver were harvested for flow cytometric analyses. **(A)** The total leukocyte numbers in the spleen and liver. **(B)** The absolute cell numbers of B cell subsets (FOB, MZB, and B1 cells) in the spleen and liver. The mean values ± SD (n = 4 in A and B; per group in the experiment; Student’s t-test; (* *p* < 0.05 and ** *p* < 0.01) are shown. One representative experiment of two experiments is shown. ns, not significant.

**
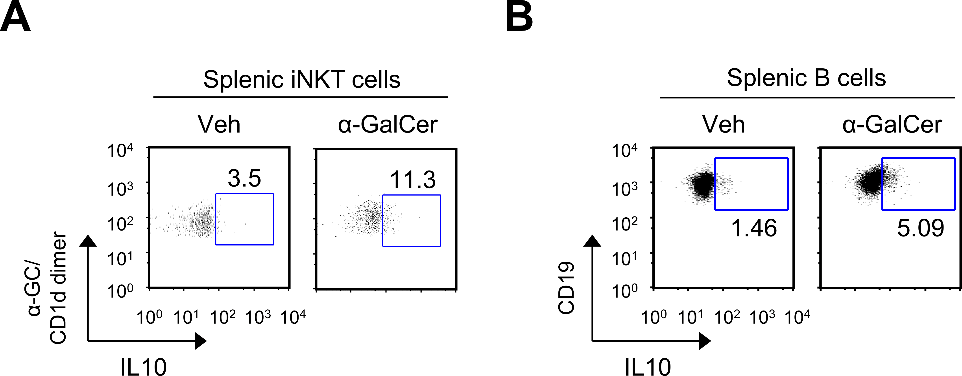
**

**Supplementary Figure 7. IL10 expression by splenic iNKT and B cells in α-GalCer-pretreated mice.**

WT B6 mice were injected i.p. with α-GalCer (2 μg/mouse), and seven days later, the spleen was harvested for flow cytometric analyses. **(A, B)** Representative FACS plots for the frequencies of IL10-expressing iNKT **(A)** and B cells **(B)**.
